# La métrica Sigma del sistema Alinity ci: estudio sobre 39 magnitudes químicas y de inmunoensayo

**DOI:** 10.1515/almed-2021-0025

**Published:** 2021-04-16

**Authors:** Fatima Zehra Kanani, Adnan Haider Kazmi, Bushra Kaleem

**Affiliations:** Sección de patología química, Departamento de Patología, The Indus Hospital, Karachi, Pakistan; Indus Hospital Research Centre, The Indus Hospital, Karachi, Pakistan

**Keywords:** especificaciones de rendimiento, exactitud, precisión, Seis Sigma, validación

## Abstract

**Objetivos:**

La métrica Sigma es una forma útil y económica de verificar la calidad de las pruebas en los laboratorios clínicos. Alinity ci es un analizador (Abbott Diagnostics) lanzado recientemente, cuyo rendimiento aún no ha sido suficientemente estudiado. Calculamos el valor Sigma de 39 magnitudes químicas y de inmunoensayo de dos sistemas Alinity ci.

**Métodos:**

Las métricas Sigma se derivaron de los estudios de validación del método. El coeficiente de variación (CV) se estimó siguiendo la guía CLSP EP 15. Se emplearon tres métodos para comprobar desviaciones: material de evaluación de rendimiento, comparación de métodos alternativos y prueba de linealidad. Se emplearon límites de error total permitido similares o inferiores a los de los estudios de referencia.

**Resultados:**

Todas las magnitudes químicas, excepto el nitrógeno ureico en sangre (BUN), mostraron un valor Sigma > 6 en uno o más niveles y métodos. Ninguna de las magnitudes estudiadas obtuvo <3 Sigma. Entre los electrolitos, el sodio obtuvo <3 Sigma en dos niveles en el método de evaluación de rendimiento, aunque alcanzó >4 Sigma en los otros dos métodos. Los niveles Sigma obtenidos fueron similares a los de estudios anteriores.

**Conclusiones:**

Los valores de Sigma fueron aceptables en todas las magnitudes químicas, de inmunoensayo y electrolitos analizados con Alinity ci. La métrica Sigma es una herramienta objetiva, económica y extendida de control interno de la calidad. Calculamos la métrica Sigma de numerosas pruebas de alto rendimiento. Es necesario evaluar el rendimiento de estas pruebas a largo plazo.

## Introducción

La métrica Sigma es una herramienta de evaluación de calidad ampliamente utilizada en la industria, cuyo uso en los laboratorios clínicos se está extendiendo [1]. Esta herramienta se basa en el concepto de los defectos por millón de oportunidades y su objetivo es minimizar los errores en los procesos [2]. En el laboratorio clínico, se suelen emplear estadísticas de control interno y externo de calidad para calcular valores mensurables para los cálculos Sigma, a partir de datos de exactitud y precisión de la prueba. Analistas clínicos y otros profesionales de laboratorio emplearon distintos métodos basados en esta técnica para evaluar y verificar el rendimiento de su laboratorio. La precisión se suele calcular mediante el coeficiente de variación generado a partir del análisis diario de los diferentes niveles de control interno de calidad empleados para las pruebas. La exactitud se estudia calculando la desviación, normalmente a partir de valores de grupos de comparación sobre esquemas de pruebas de rendimiento, aunque también se han utilizado otros métodos [3], [4]. Otra variable en el cálculo de la métrica Sigma es el límite de error total permitido (TEα). Se han propuesta diversos TEα para distintas magnitudes basados en el rendimiento analítico óptimo [5], [6], [7], [8], las recomendaciones clínicas [9] o la variación biológica [10]. En la Tabla Suplementaria S1 se muestra un resumen de algunas de las fuentes actualmente disponibles.

La principal ventaja de la métrica Sigma es que se trata de una herramienta cuantificable y mensurable, que permite la evaluación y seguimiento objetivo del rendimiento de una prueba. Seis Sigma y superiores son la clase estándar mundial, en la que hay menos de cuatro defectos por millón de oportunidades. El nivel mínimo aceptable para una prueba analítica clínica es el nivel tres Sigma. Este nivel se aproxima a los 67.000 defectos por millón de oportunidades, por debajo del cual las pruebas no se consideran seguras clínicamente para realizar análisis de muestras de pacientes [11], [12], [13].

Otra ventaja importante de calcular la métrica Sigma de las pruebas analíticas es que permite medir objetivamente la calidad de las pruebas, pudiendo así espaciar la frecuencia de los controles internos de calidad. Una prueba con una alta precisión y exactitud, que arroja valores de Sigma elevados, requiere un menor número de controles internos de calidad por serie, más muestras por serie y menos reglas Westgard que aplicar. Por otro lado, las pruebas de mayor precisión o desviación requieren más controles internos de calidad, series más pequeñas, menos muestras y más reglas Westgard que aplicar. Este es el concepto por el que aboga Westgard en su última versión de Reglas de Westgard Sigma [14], [15]. Esta herramienta reduce la carga de trabajo y los costes de las pruebas ordinarias, con un enfoque más centrado en las pruebas que suelen dar más problemas. El coste de las pruebas de laboratorio es una de sus principales limitaciones, no solo para las regiones menos desarrolladas, sino también para los laboratorios comerciales de todo el mundo. La crisis económica que está empezando a afectar a diversos países, especialmente ante la actual pandemia, ha evidenciado que las organizaciones necesitan desarrollar estrategias para reducir los costes de sus operaciones. La métrica Sigma es una de dichas estrategias.

En este estudio, empleamos tres métodos diferentes para calcular la métrica Sigma de la serie Alinity ci, en la que se han calculado las desviaciones a partir de datos de materiales de evaluación de rendimiento. Calculamos la métrica Sigma de las pruebas realizadas con el sistema Alinity ci durante las validaciones iniciales, lo cual forma parte de un ejercicio continúo orientado a adaptar el uso de controles internos de calidad.

## Materiales y métodos

Dado que el presente estudio no estaba relacionado con la experimentación en animales o seres humanos, estaba exento de aprobación por parte del Comité Ético correspondiente. Se analizaron 39 parámetros en dos analizadores Alinity c y Alinity i entre los meses de junio y septiembre de 2018. Se conectaron dos analizadores Alinity ci a un sistema de automatización a3600, incluyendo los procesos pre y postanalíticos, como parte del sistema de automatización total del laboratorio químico clínico central. Los datos se generaron durante los ejercicios de validación del método, realizados con estos analizadores.

Abbott Diagnostics lanzó al mercado la plataforma analítica Alinity en 2017. Alinity c es un analizador químico clínico de electrolitos totalmente automatizado con electrodos selectivos de ión indirecto sobre tecnología de chip integrado (ICT, por sus siglas en inglés). Por otro lado, el sistema Alinity i es un analizador de inmunoensayo basado en micropartículas paramagnéticas y detección quimioluminiscente basada en un conjugado marcado con éster de acridinio. Se incluyeron los siguientes parámetros químicos clínicos y electrolitos: albúmina, fosfatasa alcalina (ALP), alanina aminotransferasa (ALT), amilasa, aspartato aminotransferasa (AST), nitrógeno ureico en sangre (BUN), calcio, cloruro, colesterol, CO_2_, creatinina fosfoquinasa (CPK), creatinina, bilirrubina directa, gamma-glutamil transferasa (GGT), glucosa, colesterol de lipoproteínas de alta densidad (C-HDL) hierro, lactato deshidrogenasa (LDH), colesterol de lipoproteínas de baja densidad (C-LDL), magnesio, fósforo, potasio, sodio, bilirrubina total, proteína total, triglicéridos y ácido úrico. Por otro lado, los parámetros de inmunoensayo incluidos fueron alfa-fetoproteína (AFP), gonadotropina coriónica humana beta (β -HCG), triyodotironina libre (FT 3), libre tiroxina (FT 4), ferritina, hormona estimulante del folículo (FSH), hormona luteinizante (LH), prolactina, hormona estimulante del tiroides (TSH), antígeno prostático específico total (PSA) y vitaminas B12 y D. Se calculó la métrica Sigma de todas las magnitudes en los dos conjuntos de analizadores y se consideraron los valores medios para el propósito del estudio. Las métricas Sigma se calcularon aplicando la siguiente fórmula:

**Σσ** = (TEα − desviación)/CV (donde Σσ es el valor de Sigma, TEα es el error total permitido y CV es el coeficiente de variación).

A. **Error total permitido:** Todos los TEαs se extrajeron de la Ley de Mejora de Laboratorios Clínicos (CLIA), el Colegio de Patólogos Americanos (CAP) y el Programa Nacional de Educación sobre el Colesterol (NCEP), a excepción de la vitamina D, cuyo TEα se extrajo de un estudio de referencia sobre variaciones biológicas (Tabla S1) [10]. Se emplearon valores TEα similares o inferiores a los utilizados en estudios comparables [16], [17].
B. **Precisión:** Se aplicaron tres niveles de control de calidad en cinco réplicas durante cinco días en los dos sistemas Alinity ci, según las recomendaciones CLSI EP 15 [18]. Finalmente, se calculó el coeficiente de variación (CV%).
C. **Desviación:** Se emplearon tres métodos diferentes para calcular la desviación:

1.
**Evaluación de rendimiento**

Previamente, se analizó y almacenaron a −40 °C los materiales de evaluación de rendimiento aplicando entre siete y diez niveles establecidos por el Colegio de Patólogos Americanos (CAP). Estos materiales se analizaron en los analizadores Alinity c e i en tres réplicas. Los resultados se compararon con la media del grupo comparable de Abbott Architect, ya que, en el momento del estudio, Alinity aún no había sido incluido en los estudios del CAP. La fórmula empleada para calcular la desviación fue [(media objetivo – media observada/media objetivo) *100]. La desviación acumulada se obtuvo a partir de la media de todas las desviaciones.

2.
**Experimento de correlación del método**

El experimento de correlación del método lo realizaron Roche Cobas c311[(Roche Diagnostics International Ltd, Rotkreuz, Suiza) para las magnitudes biológicas], Nova 16 [(Nova Biomedical Corporation 200 Prospect Street Waltham, Massachusetts, USA) para el sodio, potasio, cloruro, y CO_2_ total], Vitros Eci [(Ortho Clinical Diagnostics, Rochester, NY) para β-HCG, ferritina, FSH, FT3, FT4, LH, prolactina, TSH y PSA total] y Elecsys e411 [(Roche Diagnostics International Ltd, Rotkreuz, Suiza) para la vitamina D, vitamin B12, alfafetoproteína, hormona paratiroidea intacta y el sistema Alinity ci]. Se analizaron 40 muestras con el método existente, y se reanalizaron en las dos horas posteriores con los dos sistemas Alinity ci.

3.
**Experimento de linealidad**

Se realizaron estudios de linealidad en todas las pruebas en tres réplicas aplicando siete niveles y empleando material de linealidad comercial (magnitudes químicas y electrolitos) (o muestras de pacientes) (inmunoensayo), exceptuando las magnitudes con calibradores multipunto. La desviación se calculó a partir de la diferencia entre los valores esperados y la media de los valores observados, tal como se ha indicado anteriormente. Las desviaciones y valores de Sigma obtenidos en el experimento de linealidad se muestran en la Tabla Suplementaria S2.

## Resultados

En la Tabla 1 se pueden observar los niveles de concentración, precisión, coeficientes de variación y desviación de estas magnitudes. Como se puede ver, la precisión de todas las magnitudes es igual o inferior al 5,0%, mientras que existe variabilidad en las desviaciones obtenidas con los distintos métodos. La precisión para las magnitudes químicas y los electrolitos supera a la de los inmunoensayos.

**Tabla 1:** CV y desviación de magnitudes según dos métodos.

Magnitudes	CV, %			Desviación, %	
	L1	L2	L3	Método de evaluación de rendimiento	Comparación de métodos alternativos
**Analitos químicos clínicos**					
ALB	1,062	0,835	0,599	3,49	1,41
ALP	2,202	1,623	1,413	5,34	0,29
ALT	1,687	1,776	0,966	2,78	−0,53
AMI	2,289	0,913	0,694	3,59	−1,07
AST	1,628	1,046	0,876	5,52	−0,64
BUN	1,808	1,923	1,548	−1,77	1,16
Ca	1,221	0,961	0,967	−0,75	−0,27
COL	1,030	0,980	0,990	−0,47	−0,46
CO_2_	3,204	3,507	4,871	1,22	1,94
CPK	1,273	0,740	0,691	14,01	1,86
CREA	3,109	1,726	0,977	2,38	2,98
BD	1,512	2,490	1,172	−0,40	0,85
GGT	1,886	0,908	1,002	9,57	−1,20
GLU	0,975	0,538	0,335	1,15	−0,34
HDL-C	1,675	0,791	0,962	4,21	−1,65
Hierro	1,052	0,627	0,910	3,18	−2,36
LDH	1,042	0,988	0,781	4,27	−0,50
LDL-C	0,950	0,643	1,435	1,31	−1,75
Mg	1,996	1,719	1,725	−2,85	−1,21
P	2,028	1,337	0,974	−0,69	−0,22
BT	0,916	2,195	2,174	0,63	−0,17
TG	2,145	0,943	0,853	−3,13	2,16
PT	0,725	0,772	0,689	0,47	−0,03
AU	2,124	0,922	0,814	−0,43	−0,42
**Inmunoensayo**					
AFP	3,351	3,123	3,230	−0,26	−1,62
βHCG	4,382	4,158	2,198	−4,35	0,77
FER	2,839	2,725	2,606	−5,65	−10,53
FSH	1,890	3,047	2,510	−0,05	−3,49
T3 libre	2,744	3,420	3,164	−6,64	−3,33
T4 libre	2,973	1,239	2,513	−5,83	1,79
LH	2,063	2,762	2,360	6,50	−4,63
Prolactina	2,241	2,667	2,667	3,64	3,11
PSA total	1,370	1,910	2,274	−3,46	−7,30
TSH	3,157	4,085	2,767	6,14	−2,22
VIT B12	5,061	3,709	4,640	−2,93	−0,07
VIT D	4,833	4,952	3,337	−3,90	−6,40
**Electrolitos**					
Cl	0,647	0,706	0,583	1,51	1,42
K	1,856	1,173	0,842	0,43	0,41
Na	0,766	0,537	0,737	1,58	−0,07

ALB, albúmina; ALP, fosfatasa alcalina; ALT, alanina aminotransferasa; AMI, amilasa; AST, aspartato aminotransferasa; Ca, calcio; COL, colesterol; CO_2_, dióxido de carbono; CPK, creatinina fosfoquinasa; CREA, creatinina; BD, bilirrubina directa; GGT, gamma glutamil transferasa; GLU, glucosa; HDL, lipoproteína de alta densidad; LDH, lactato deshidrogenasa; LDL, lipoproteína de baja densidad; Mg, magnesio; P, fósforo; PSA total, antígeno prostático específico total; BT, bilirrubina total; TG, triglicéridos; PT, proteína total; AU, ácido úrico; AFP, alfa fetoproteína; βHCG, gonadotropina coriónica humana beta; FER, ferritina; FSH, hormona estimulante del folículo; T3 libre, triyodotironina libre; T4 libre, tiroxina libre; HL, hormona luteinizante; TSH, hormona estimulante de la tiroides; VIT B12, vitamina B12; VIT D, vitamina D; Cl, cloruro; K, potasio; Na, sodio.

La Tabla 2 incluye un análisis de las métricas Sigma. Con respecto a los análisis clínicos, todos los parámetros excepto BUN, mostraron valores de Sigma >6 en uno o más niveles. Cabe señalar que se obtuvieron valores de Sigma <6 en uno o más niveles o métodos en BUN, CO_2_, creatinina, fósforo y triglicéridos. Con respecto a los inmunoensayos, se obtuvieron valores de Sigma >6 en todos los niveles y con todos los métodos para la ferritina, prolactina y PSA, mientras que en otros parámetros se obtuvieron valores de Sigma >6 en uno o más niveles en los diferentes métodos. No se obtuvo un valor de Sigma <3 en ninguno de los parámetros de las analíticas clínicas. En relación a los electrolitos, el potasio obtuvo un valor Sigma >6 en todos los niveles y métodos, mientras que el sodio fue el único parámetro con un valor de Sigma <3 en dos niveles en el método de evaluación de rendimiento (Tabla 2).

**Tabla 2:** Análisis de métrica de Sigma de magnitudes mediante varios métodos utilizando Alinity ci.

Magnitudes	TEα	FUENTE	Método de rendimiento			Comparación de métodos alternativos		
			L1	L2	L3	L1	L2	L3
**Química clínica**								
ALB	10%	CLIA	6,13	7,79	10,86	8,09	10,29	14,33
ALP	30%	CLIA	11,20	15,19	17,45	13,49	18,30	21,03
ALT	20%	CLIA	10,21	9,70	17,84	11,54	10,96	20,16
AMI	30%	CLIA	11,54	28,92	38,07	12,64	31,67	41,69
AST	20%	CLIA	8,89	13,84	16,52	11,89	18,50	22,10
BUN	9%	CLIA	4,00	3,76	4,67	4,34	4,08	5,06
Ca	9,72%	CLIA	7,34	9,33	9,28	7,74	9,83	9,78
COL	9%	NCEP	8,28	8,80	8,62	8,29	8,71	8,63
CO_2_	25%	CAP	7,42	6,78	4,88	7,20	6,57	4,73
CPK	30%	CLIA	12,56	21,60	23,12	22,11	38,01	40,70
CREA	15%	CLIA	4,06	7,32	12,93	3,87	6,97	12,31
DB	20%	CLIA	12,96	7,87	16,72	12,67	7,69	16,34
GGT	22,1%	RICOS	6,65	13,80	12,50	11,08	23,01	20,85
GLU	10%	CLIA	9,08	16,45	26,42	9,91	17,95	28,83
HDL-C	30%	CLIA	15,40	32,61	26,80	16,92	35,85	29,46
Hierro	20%	CLIA	15,99	26,84	18,49	16,76	28,14	19,38
LDH	20%	CLIA	15,10	15,92	20,15	18,71	19,74	24,98
LDL-C	20%	CAP	19,69	29,09	13,02	19,22	28,40	12,72
Mg	25%	CLIA	11,09	12,88	12,84	11,92	13,84	13,79
P	10,7%	CAP	4,94	7,48	10,28	5,17	7,87	10,77
BT	20%	CLIA	21,14	8,83	8,91	21,63	9,03	9,12
GT	15%	NCEP	5,54	12,58	13,91	5,99	13,61	15,04
PT	10%	CLIA	13,15	12,35	13,83	13,76	12,92	14,46
AU	17%	CLIA	7,80	17,98	20,36	7,80	17,99	20,38
**Inmunoensayo**								
AFP	20%	RCPA	5,89	6,32	6,11	5,49	5,88	5,69
βHCG	30%	RiliBAK	5,85	6,17	11,67	6,67	7,03	13,30
FER	30%	CAP	8,57	8,94	9,34	6,86	7,15	7,47
FSH	20%	RCPA	10,55	6,55	7,95	8,73	5,42	6,58
T3 libre	17%	RICOS	3,78	3,03	3,27	4,98	4,00	4,32
T4 libre	16%	Spanish EQA Minimum	3,42	8,21	4,05	4,78	11,47	5,66
LH	20%	RCPA	6,54	4,59	5,72	7,45	5,57	6,51
Prolactina	20%	RCPA	7,30	6,14	6,13	7,54	6,33	6,33
PSA total	20%	Ricos Desirable	12,07	8,66	7,27	11,98	8,59	7,22
TSH	23,7%	CLIA	8,70	6,72	9,92	6,81	5,26	7,76
VIT B12	30%	WSLH	5,35	7,30	5,83	5,91	8,07	6,45
VIT D	30%	Biological Variation Paper	5,40	5,27	7,82	4,88	4,76	7,07
**Electrolitos**								
Cl	5%	CLIA	5,39	4,94	5,98	5,53	5,06	6,13
K	17,97%	CLIA	9,45	14,96	20,84	9,46	14,97	20,86
Na	3,57%	CLIA	2,59	3,70	2,70	4,57	6,52	4,75

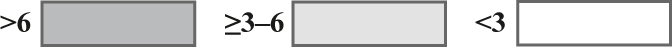
 ALB, albúmina; ALP, fosfatasa alcalina; ALT, alanina aminotransferasa; AMI, amilasa; AST, aspartato aminotransferasa; Ca, calcio; COL, colesterol; CO_2_, dióxido de carbono; CPK, creatinina fosfoquinasa; CREA, creatinina; BD, bilirrubina directa; GGT, gamma glutamil transferasa; GLU, glucosa; HDL, lipoproteína de alta densidad; LDH, lactato deshidrogenasa; LDL, lipoproteína de baja densidad; Mg, magnesio; P, fósforo; PSA total, antígeno prostático específico total; BT, bilirrubina total; TG, triglicéridos; PT, proteína total; AU, ácido úrico; AFP, alfa fetoproteína; βHCG, gonadotropina coriónica humana beta; FER, ferritina; FSH, hormona estimulante del folículo; T3 libre, triyodotironina libre; T4 libre, tiroxina libre; HL, hormona luteinizante; TSH, hormona estimulante de la tiroides; VIT B12, vitamina B12; VIT D, vitamina D; Cl, cloruro; K, potasio; Na, sodio. CLIA 1992 fue la fuente de errores totales permitidos para distintas magnitudes.

**Figura 1:**
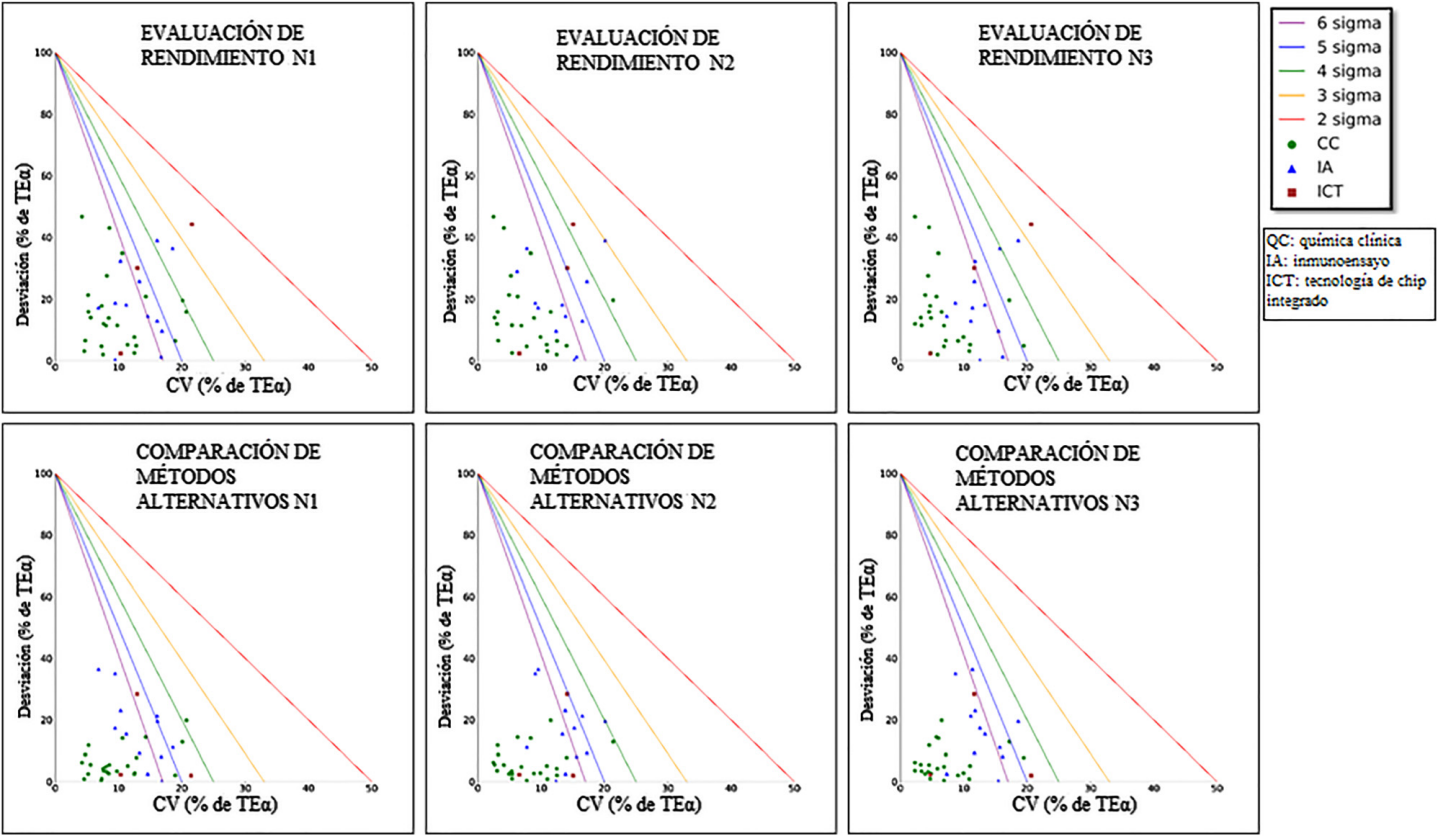
Gráficos de decisión de método normalizados en los que se muestran las métricas de Sigma empleando dos métodos diferentes.

Tal como se muestra en la Tabla 3, los valores de Sigma obtenidos en este estudio son comparables a los documentados en otros estudios sobre Alinity. En las Figuras 1 y S1 se detallan las Method Decision Charts (MEDx) realizadas con las librerías Matplotlib en Python3.6 en tres niveles de concentración con los tres métodos.

**Tabla 3:** Comparación de estudios sobre el sistema Alinity ci.

Magnitudes	Westgard [17]				Taher [16]				Estudio actual, 2019			
	TEα, %	Fuente	Conc,	σ	TEα, %	Fuente	Conc.	σ	TEα, %	Fuente	Conc.	σ
**Química clínica**												
ALB	10	CLIA	2,91 g/dL	20,6	–	–		–	10	CLIA	2.99 g/dL	6.13
ALP	30	CLIA	187,25 U/L	16,7	30	CLIA	151 U/L	10,6	30	CLIA	193,02 U/L	15,19
ALT	20	CLIA	30,2 U/L	10,8	20	CLIA	28 U/L	6	20	CLIA	26,08 U/L	10,21
AMI	30	CLIA	114,32 U/L	38,5	30	CLIA	132 U/L	27	30	CLIA	115,12 U/L	28,92
AST	20	CLIA	41,93 U/L	11,4	20	CLIA	40 U/L	8	20	CLIA	41,76 U/L	8,89
BUN	9	CLIA	38,82 mg/dL	4,24	9	CLIA	48,4 mg/dL	5,7	9	CLIA	43,22 mg/dL	3,76
Ca	9,72	CLIA	10,29 mg/dL	8,3	9,72	CLIA	9,82 mg/dL	5,3	9,72	CLIA	9,89 mg/dL	9,33
COL	10	CLIA	154,99 mg/dL	9,06	–	–	–	–	9	NCEP	185,86 mg/dL	8,70
CO_2_	25	CAP	22,34 mEq/L	5,6	25	CAP	21 mEq/L	3,9	25	CAP	21,27 mEq/L	6,78
CPK	30	CLIA	148,21 U/L	26,2	–	–	–	–	30	CLIA	233,34 U/L	21,60
CREA	15	CLIA	2,00 mg/dL	6,9	–	–	–	–	15	CLIA	2,12 mg/dL	7,32
DB	44,5	Ricos deseable	0,4 mg/dL	11,2	–	–	–	–	20	CLIA	0,41 mg/dL	12,96
GGT	22,1	Ricos deseable	71,42 U/L	15,7	–	–	–	–	22,1	RICOS	59,94 U/L	13,80
GLU	10	CLIA	126,86 mg/dL	8,7	10	CLIA	118,8 mg/dL	5,7	10	CLIA	141,76 mg/dL	16,45
HDL	30	CLIA	51,77 mg/dL	13,6	–	–	–	–	30	CLIA	41,08 mg/dL	15,40
Hierro	20	CLIA	103,03 μg/dL	14,4	–	–	–	–	20	CLIA	99,72 μg/dL	15,99
LDH	20	CLIA	127,98 U/L	5,5	–	–	–	–	20	CLIA	129,10 U/L	15,10
LDL	20	CAP	78,44 mg/dL	11,7	–	–	–	–	20	CAP	63,04 mg/dL	19,69
Mg	25	CLIA	2,22 mg/dL	17,5	25	CLIA	1,87 mg/dL	16,7	25	CLIA	2,32 mg/dL	12,88
P	10,7	CAP	4,24 mg/dL	5,8	10,7	CAP	–	5,4	10,7	CAP	4,37 mg/dL	7,48
BT	20	CLIA	2,78 mg/dL	9,14	20	CLIA	2,81 mg/dL	7,6	20	CLIA	2,57 mg/dL	8,83
GT	25	CLIA	150,79 mg/dL	27,6	–	–	–	–	15	NCEP	142,2 mg/dL	12,58
PT	10	CLIA	5,09 g/dL	7,65	10	CLIA	4,9 g/dL	6,2	10	CLIA	4,48 g/dL	13,15
AU	17	CLIA	2,49 mg/dL	15,6	–	–	–	–	17	CLIA	2,34 mg/dL	7,80
**Inmunoensayo**												
AFP	–	–		–	–	–	–	–	20	RCPA	5,42 IU/L	5,89
βHCG	30	RiliBAK	24,45 IU/mL	5,48	30	RiliBAK	26 IU/L	4,4	30	RiliBAK	4,49 IU/L	5,85
FER	–	–	–	–	–	–	–	–	30	CAP	32,83 ng/mL	8,57
FSH	–	–	–	–	–	–	–	–	20	RCPA	35,73 U/L	7,95
T3 libre	17	Mínimo Ricos	6,15 pg/mL	3,12	–	–	–	–	17	RICOS	5,08 pg/mL	3,78
T4 libre	16	Minimo EQA en España Mínimo	1,16 ng/dL	5,94	–	–	–	–	16	Minimo EQA en España	11,34 ng/dL	3,42
LH	–	–		–	–	–	–	–	20	RCPA	38,43 U/L	5,72
Prolactina	29,4	Ricos deseable	38,75 ng/mL	8,83	–	–	–	–	20	RCPA	41,44 ng/mL	6,13
TSH	23,7	Ricos deseable	0,31 μIU/mL	10,9	23,7	Ricos deseable	0,16 µIU/mL	10	23,70	Ricos deseable	0,051 μIU/mL	5,56
PSA^a^	33,6	Ricos deseable	0,39 ng/mL	5,88	–	–	–	–	20	CLIA	3,40 ng/mL	8,66
VIT B12	–	–		–	–	–	–	–	30	WSLH	294,68 pg/mL	5,35
VIT D	30	Estudio de variación biológica	21,14 ng/mL	7,17	–	–	–	–	30	Estudio de variación biológica	23,28 ng/mL	5,27
**Electrolitos**												
Cl	5	CLIA	94,65 mmol/L	6,57	5	CLIA	96 mmol/L	5,2	5	CLIA	96,73 mmol/L	4,94
K	17,97	CLIA	2,78 mmol/L	11,9	18	CLIA	2,7 mmol/L	11,8	17,97	CLIA	2,64 mmol/L	9,45
Na	3,57	CLIA	112,03 mmol/L	4,2	4	CLIA	121 mmol/L	4,1	3,57	CLIA	121,95 mmol/L	2,59

ALB, albúmina; ALP, fosfatasa alcalina; ALT, alanina aminotransferasa; AMI, amilasa; AST, aspartato aminotransferasa; Ca, calcio; COL, colesterol; CO_2_, dióxido de carbono; CPK, creatinina fosfoquinasa; CREA, creatinina; BD, bilirrubina directa; GGT, gamma glutamil transferasa; GLU, glucosa; HDL, Lipoproteína de alta densidad; LDH, lactato deshidrogenasa; LDL, Lipoproteína de baja densidad; Mg, magnesio; P, fósforo; PSA total, antígeno prostático específico total; BT, bilirrubina total; TG, triglicéridos; PT, proteína total; AU, ácido úrico; AFP, alfa fetoproteína; βHCG, gonadotropina coriónica humana beta; FER, ferritina; FSH, hormona estimulante del folículo; T3 libre, triyodotironina libre; T4 libre, tiroxina libre; HL, hormona luteinizante; TSH, hormona estimulante de la tiroides; VIT B12, vitamina B12; VIT D, vitamina D; Cl, cloruro; K, potasio; Na, sodio. CLIA 1992 fuente de los errores totales permitidos para distintas magnitudes. ^a^Westgard había analizado el PSA libre, mientras que en el presente estudio se ha analizado el PSA total, resultando en diferentes concentraciones. De este modo, es imposible la comparación.

## Discusión

La métrica Sigma es una herramienta sencilla y objetiva para el seguimiento y cuantificación de la calidad de las pruebas de laboratorio. Estos valores se pueden calcular fácilmente a partir de los valores de los controles de calidad ordinarios generados en los laboratorios, sin que ello suponga ningún coste adicional. Además, se puede utilizar para diseñar la política de control interno de calidad y reducir la frecuencia y número de controles diarios, lo que supone un ahorro en costes. Ante la actual crisis económica mundial, en la que los laboratorios tratan de alcanzar un equilibrio entre la aplicación de medidas de control de calidad y los costes que ello implica, ha surgido una nueva aplicación de la métrica Sigma basada en reglas de control de calidad.

Nuestros estudios iniciales de validación en Alinity ci (Abbott Diagnostics, Abbott Gmbh & Co. KG, Wiesbaden, Germany) han arrojado resultados prometedores y muestran valores de Sigma elevados para la mayoría de las pruebas. Los tres métodos mostraron un buen rendimiento, aunque se obtuvieron resultados inferiores con el método de evaluación de rendimiento. Esto podría deberse a que las muestras se almacenaron a −40 °C previamente a su análisis. Para compensar esta limitación, primero se confirmó la integridad de la muestra analizando las muestras en los sistemas existentes y comparando los resultados con los resultados resumidos del paciente. A continuación, se volvieron a analizar en un margen inferior a las dos horas en el sistema Alinity ci. Aunque para la obtención de valores de Sigma se realizaron una evaluación de rendimiento, una comparación de métodos alternativos y estudios de linealidad, recomendamos usar la evaluación de rendimiento como la herramienta más estandarizada para comparar de forma continuada el método de un laboratorio con los de otros laboratorios de cualquier parte del mundo. La comparación de métodos alternativos resulta útil durante la fase de evaluación inicial, aunque a veces resulta difícil emplearla en los inmunoensayos, aunque los estudios de linealidad son más útiles a la hora de medir rangos, así como en los estudios de verificación y calibración, que para calcular desviaciones.

La elección del error total permitido es el principal determinante a la hora de calcular valores de Sigma [19]. Cuando más amplio sea el límite, mayor será el valor de Sigma, causando una falsa sensación de seguridad y derivando en una sobreestimación de la calidad de la prueba. El empleo de límites más exigentes reduce los niveles de Sigma, pero otorga una imagen más veraz de la calidad de la prueba. Hemos tratado de emplear TEα similares o inferiores a los utilizados en estudios comparativos sobre Alinity ci. Westgard [17] y Taher et al. [16] realizaron una secuencia en la que utilizaron los límites de error más bajos que se pueden obtener en las pruebas en un contexto real. Aplicamos los mismos límites, con el fin de comparar la escala Sigma obtenida con los análisis iniciales realizados según especificaciones factoriales, o sobre el campo por los investigadores de la compañía. En algunas pruebas como la bilirrubina directa (20% frente a 44.5%), triglicéridos (15% frente a 25%) y prolactina (20% frente a 29.4%), elegimos unos límites de error permitido inferiores a los seleccionados en los estudios de referencia, obteniendo aún así el nivel seis Sigma. Los objetivos de error total permitido propuestos por CLIA en 2019 son un paso más para elevar el listón, y es nuestra intención emplearlos en futuros estudios.

Otro elemento del presente estudio a tener en cuenta es el número de magnitudes incluidas. Así, se incluyeron múltiples magnitudes que no fueron analizadas en los estudios previos de Westgard et al. [17] (AFP, ferritina, FSH, LH y vitamina B12) [16] (albúmina, colesterol, CPK, creatinina, bilirrubina directa, GGT, HDL, hierro, LDH, LDL, triglicéridos, ácido úrico, AFP, ferritina, FSH, FT3, FT4, LH, prolactina, PSA, vitaminas B12 y D).

Los analitos de química clínica suelen mostrar valores de Sigma más altos que los parámetros de inmunoensayo. Esto se debe a la mayor precisión de estas pruebas, en comparación con los inmunoensayos. La variabilidad de los niveles Sigma entre los diferentes métodos se debe a la variación en las desviaciones generadas, ya que la precisión empleada en los cálculos fue constante, ya que se obtuvo a partir de datos de controles internos de calidad.

El presente estudio presenta algunas limitaciones. En primer lugar, los resultados de las evaluaciones de rendimiento se compararon con los obtenidos en grupos de comparación de Architect debido al hecho de que no se disponía de los resultados de participación del CAP sobre Alinity en el momento de la realización de este estudio. En segundo lugar, este estudio se realizó en un periodo corto de tiempo con EP15 para la generación de datos de precisión y empleando un único lote de reactivos, calibradores y controles. La experiencia actual tras la implementación de estas pruebas ha cambiado completamente, disponiendo de análisis de grupos de comparación en tiempo real en encuestas del CAP, y con distintos lotes de reactivos, calibradores, controles, condiciones ambientales y operadores.

## Conclusiones

El sistema Alinity ci generó una métrica Sigma aceptable para todos los analitos químicos clínicos y de inmunoensayo habitualmente utilizados en la práctica clínica. La métrica Sigma es una herramienta útil para evaluar la calidad de una magnitud biológica basándonos en su precisión y exactitud, que permitirá a los laboratorios modificar y espaciar la frecuencia de los controles internos de calidad. También permitirá a los laboratorios reducir costes sin comprometer la seguridad del paciente. En este estudio se aplicaron diferentes límites de error total aceptable. No obstante, con la introducción de los objetivos de error total permitido de CLIA de 2019, proponemos que estos sean adoptados por todos los laboratorios para calcular sus métricas Sigma.

## Supplementary Material

Supplementary Material Details

## Material Suplementario

La versión en línea de este artículo ofrece material suplementario (https://doi.org/10.1515/almed-2021-0025).
