# Supplementary material for: La métrica Sigma del sistema Alinity ci: estudio sobre 39 magnitudes químicas y de inmunoensayo
Source: Adv Lab Med. 2021 Apr 16;2(2):277–85. [Article in Spanish] doi: 10.1515/almed-2021-0025 (PMC10197295; doi:10.1515/almed-2021-0025)

**MATERIAL SUPLEMENTARIO**

**Tabla S1: Variedad de fuentes de error total permitido (TEαs)**

| MAGNITUD | CLIA | | CAP | RCPA | RICOS | | RiliBÄK | Mínimo EQA en España | NCEP | ESTUDIO DE VARIABILIDAD BIOLÓGICA |
| --- | --- | --- | --- | --- | --- | --- | --- | --- | --- | --- |
|  | **2019** | **1992** |  |  | **DESEABLE** | **ÓPTIMO** |  |  |  |  |
| QUÍMICA CLÍNICA | | | | | | | | | | |
| ALB | 8% | 10% | 10% | 6% | 4,07% | 23,3% | - | 14% | - | - |
| ALP | 20% | 30% | 30% | 12% | 12,04% | - | 18% | 31% | - | - |
| ALT | 15% | 20% | 20% | 12% | 27,48% | 13,7% | 21% | 23% | - | - |
| AMI | 10% | 30% | 30% | 10% | 14,6% | 7,3% | - | 35% | - | - |
| AST | - | 20% | 20% | 12% | 16,69% | 8,3% | - | 21% | - | - |
| BUN | 9% | 9% | 9% | 12% | 15,5% | 7,8% | 20% | 19% | - | - |
| Ca | 9,72% | 9,72% | 1mg/Dl. | 4% | 2,4% | - | 10% | 11% | - | - |
| COL | 10% | 10% | 10% | 6% | 9,01% | 4,5% | 13% | 11% | 9% | - |
| CO_2_ | 20% | - | 25% | 8% | 5,7% | - | 12% | - | - | - |
| CPK | 20% | 30% | 30% | 12% | 30,3% | 15,2% | 20% | 24% | - | - |
| CREA | 10% | 15% | 15% | 8% | 8,87% | 7,7% | 20% | 20% | - | - |
| DB | - | 20% | 20% | 20% | 44,5% | - | - | - | - | - |
| GGT | 15% | 20% | 3SD | 12% | 22,1% | 11,1% | 21% | 22% | - | - |
| GLU | 8% | 10% | 10% | 8% | 6,96% | - | 15% | 11% | - | - |
| HDL-C | 20% | 30% | 30% | 12% | 11,63% | - | - | 33% | 13% | - |
| HIERRO | 15% | 20% | 20% | 12% | 30,7% | 15,3% | - | 24% | - | - |
| LDH | 15% | 20% | 20% | 8% | 11,4% | 5,7% | 18% | 26% | - | - |
| LDL-C | 20% | - | 20% | 10% | 11,9% | - | - | - | 12% | - |
| Mg | 15% | 25% | 25% | 8% | 4,8% | - | 15% | - | - | - |
| P | 10% | - | 10,7% | 8% | 10,11% | 5,1% | 16% | 17% | - | - |
| BT | 20% | 20% | 20% | 12% | 26,94% | 13,5% | 22% | 24% | - | - |
| GT | 15% | 25% | 25% | 12% | 28% | 13% | 16% | 18% | 15% | - |
| PT | 8% | 10% | 10% | 5% | 3,63% | - | 10% | 12% | - | - |
| AU | 10% | 17% | 17% | 8% | 11,9% | 6% | 13% | 17% | - | - |
| INMUNOENSAYO | | | | | | | | | | |
| AFP | 20% | 3SD | 3SD | 20% | 21,9% | 10,9% | - | - | - | - |
| βHCG | 18% | 3SD | 3SD | 15% | - | - | 30% | - | - | - |
| FER | 20% | - | 30% | 15% | - | 8,4% | 25% | 21% | - | - |
| FSH | 18% | - | - | 20% | 21,19% | 10,6% | 21% | 14% | - | - |
| T3 libre | 30% | 3SD | 3SD | 20% | 11,3% | 17% | 20% | 24% | - | - |
| T4 libre | 15% | 3SD | 20% | 12% | 8,0% | - | 20% | 16% | - | - |
| LH | 20% | - | - | 20% | 27,92% | 14% | - | 17% | - | - |
| PROLACTINA | 20% | - | - | 20% | 29,4% | 14,7% | - | 22% | - | - |
| PSA TOTAL | 20% | - | - | 8% | 33,6% | 16,8% | 25% | 17% | - | - |
| TSH | 20% | 3SD | 3SD | 20% | 23,7% | 11,9% | - | 15% | - | - |
| VIT B12 | 25% | 30% | 3SD | 15% | - | - | - | - | - | - |
| VIT D | - | - | - | 15% | - | - | - | - | - | 30% |
| ELECTROLITOS | | | | | | | | | | |
| Cl | 5% | 5% | 5% | 3% | 1,5% | - |  | 9% | - | - |
| K | 0,3mmol/L | 17,97% | - | 5% | 5,61% | - |  | 8% | - | - |
| Na | 3,57% | 3,57% | - | 2% | 0,73% | - |  | 5% | - | - |

ALB- Albúmina; ALP- Fosfatasa alcalina; ALT-alanina aminotransferasa; AMI- amilasa; AST- Aspartato aminotransferasa; Ca- Calcio; COL- colesterol; CO2- Dióxido de carbono; CPK-creatinina fosfoquinasa; CREA- Creatinina; BD- Bilirrubina directa; GGT-gamma glutamil transferasa; GLU- glucosa; Lipoproteína de alta densidad HDL; LDH-lactato deshidrogenasa; Lipoproteína de baja densidad LDL; Mg-magnesio; P-fósforo; PSA TOTAL: antígeno prostático específico total; BT- Bilirrubina total; TG-triglicéridos; PT- Proteína total; AU-ácido úrico; AFP-alfa fetoproteína; βHCG-gonadotropina coriónica humana beta; FER-ferritina; FSH- Hormona estimulante del folículo; T3 libre- Triyodotironina libre; T4 libre- Tiroxina libre; HL- hormona luteinizante; TSH-hormona estimulante de la tiroides; VIT B12-Vitamina B12; VIT D- Vitamina D; Cl-Cloruro K-Potasio; Na-Sodio;

Las celdas grises indican el TEα que se tuvo en cuenta en el presente estudio.

**Tabla S2: Desviación y métrica Sigma de las magnitudes según el método de linealidad**

| **MAGNITUD** | **DESVIACIÓN** | **TEα** | **FUENTE** | **MÉTRICA SIGMA** | | |
| --- | --- | --- | --- | --- | --- | --- |
|  |  |  |  | **L1** | **L2** | **L3** |
| **ALB** | -0,63 | 10% | CLIA | 8,83 | 11,23 | 15,64 |
| **ALP** | -0,18 | 30% | CLIA | 13,54 | 18,37 | 21,10 |
| **ALT** | 0,06 | 20% | CLIA | 11,82 | 11,22 | 20,65 |
| **AMI** | -0,06 | 30% | CLIA | 13,08 | 32,78 | 43,15 |
| **AST** | -0,02 | 20% | CLIA | 12,27 | 19,10 | 22,81 |
| **BUN** | -0,14 | 9% | CLIA | 4,90 | 4,61 | 5,72 |
| **Ca** | -0,42 | 9,72% | CLIA | 7,62 | 9,68 | 9,62 |
| **COL** | 0,20 | 9% | NCEP | 8,55 | 8,98 | 8,89 |
| **CO2** | 0,47 | 25% | CAP | 7,66 | 7,00 | 5,04 |
| **CPK** | 0,66 | 30% | CLIA | 23,05 | 39,63 | 42,43 |
| **CREA** | 0,00 | 15% | CLIA | 4,82 | 8,69 | 15,36 |
| **DB** | 0,49 | 20% | CLIA | 12,91 | 7,84 | 16,65 |
| **GGT** | 1,30 | 22,1% | RICOS | 11,03 | 22,90 | 20,75 |
| **GLU** | 0,15 | 10% | CLIA | 10,10 | 18,29 | 29,38 |
| **HDL-C** | -0,57 | 30% | CLIA | 17,57 | 37,21 | 30,58 |
| **HIERRO** | 0,02 | 20% | CLIA | 18,99 | 31,88 | 21,96 |
| **LDH** | -0,06 | 20% | CLIA | 19,13 | 20,17 | 25,53 |
| **LDL-C** | -0,33 | 20% | CAP | 20,72 | 30,62 | 13,71 |
| **Mg** | -1,77 | 25% | CLIA | 1164 | 13,51 | 13,46 |
| **P** | -0,08 | 10,7% | CAP | 5,24 | 7,94 | 10,91 |
| **BT** | -0,72 | 20% | CLIA | 21,04 | 8,79 | 8,87 |
| **GT** | -0,10 | 15% | NCEP | 6,95 | 15,79 | 17,45 |
| **PT** | 0,28 | 10% | CLIA | 13,41 | 12,59 | 14,10 |
| **AU** | 0,53 | 17% | CLIA | 7,75 | 17,87 | 20,25 |
| **INMUNOENSAYO** | | | | | | |
| **AFP** | 0,00* | 20% | RCPA | 5,97 | 6,40 | 6,19 |
| **βHCG** | 0,00* | 30% | RiliBAK | 6,85 | 7,22 | 13,65 |
| **FER** | 1,20 | 30% | CAP | 10,14 | 10,57 | 11,05 |
| **FSH** | 2,05 | 20% | RCPA | 9,50 | 5,89 | 7,15 |
| **T3 libre** | 0,00* | 17% | RICOS | 6,20 | 4,97 | 5,37 |
| **T4 libre** | 0,00* | 16% | EQA Mínima en España | 5,38 | 12,92 | 6,37 |
| **LH** | 0,00* | 20% | RCPA | 9,69 | 7,24 | 8,48 |
| **PROLACTINA** | 0,34 | 20% | RCPA | 8,77 | 7,37 | 7,37 |
| **PSA TOTAL** | 0,63 | 20% | Ricos deseable | 14,14 | 10,14 | 8,52 |
| **TSH** | 2,02 | 23,7% | CLIA | 6,87 | 5,31 | 7,84 |
| **VIT B12** | 0,00* | 30% | WSLH | 5,93 | 8,09 | 6,47 |
| **VIT D** | 0,00* | 30% | Eastudio de variación biológica | 6,21 | 6,06 | 8,99 |
| **ELECTROLITOS** | | | | | | |
| **Cl** | 0,00 | 5% | CLIA | 7,72 | 7,08 | 8,57 |
| **K** | 0,02 | 17,97% | CLIA | 9,67 | 15,30 | 21,33 |
| **Na** | -0,43 | 3,57% | CLIA | 4,10 | 5,85 | 4,27 |

**>6 ≥3-6 <3**

ALB- Albúmina; ALP- Fosfatasa alcalina; ALT-alanina aminotransferasa; AMI- amilasa; AST- Aspartato aminotransferasa; Ca- Calcio; COL- colesterol; CO2- Dióxido de carbono; CPK-creatinina fosfoquinasa; CREA- Creatinina; BD- Bilirrubina directa; GGT-gamma glutamil transferasa; GLU- glucosa; Lipoproteína de alta densidad HDL; LDH-lactato deshidrogenasa; Lipoproteína de baja densidad LDL; Mg-magnesio; P-fósforo; PSA TOTAL: antígeno prostático específico total; BT- Bilirrubina total; TG-triglicéridos; PT- Proteína total; AU-ácido úrico; AFP-alfa fetoproteína; βHCG-gonadotropina coriónica humana beta; FER-ferritina; FSH- Hormona estimulante del folículo; T3 libre- Triyodotironina libre; T4 libre- Tiroxina libre; HL- hormona luteinizante; TSH-hormona estimulante de la tiroides; VIT B12-Vitamina B12; VIT D- Vitamina D; Cl-Cloruro K-Potasio; Na-Sodio; CLIA 1992 fuente de los errores totales permitidos para distintas magnitudes

*Magnitudes con calibradores multipunto

**Figura S1: Gráfica de decisión de método normalizado con los valores Sigma obtenidos en el método de linealidad**


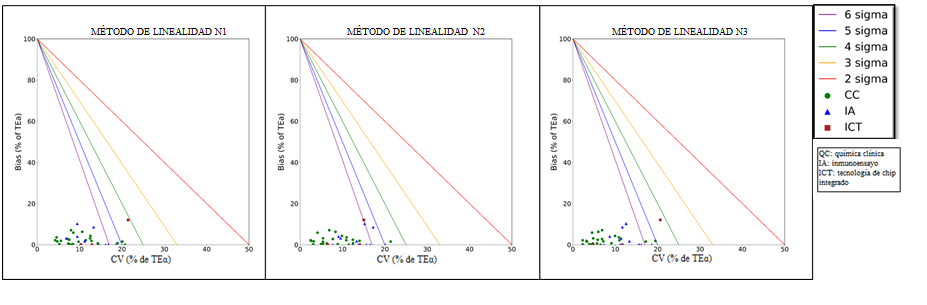

Supplement: Supplementary file 1 — Supplementary Material Details [file j_almed-2021-0025_suppl.docx]
